# Characteristics of “do not resuscitate” orders among elderly patients receiving mechanical ventilation in the intensive care unit in Taiwan

**DOI:** 10.3389/fmed.2024.1373726

**Published:** 2024-05-23

**Authors:** Pei-Jun Chen, Chung-Han Ho, Ying-Jia Lin, Ming-Hung Chang, Kuang-Ming Liao

**Affiliations:** ^1^Department of Nursing, Nantou Hospital, Ministry of Health and Welfare, Nantou, Taiwan; ^2^Department of Medical Research, Chi Mei Medical Center, Tainan, Taiwan; ^3^Department of Information Management, Southern Taiwan University of Science and Technology, Tainan, Taiwan; ^4^Cancer Center, Taipei Municipal Wanfang Hospital, Taipei Medical University, Taipei, Taiwan; ^5^Department of Internal Medicine, Chi Mei Medical Center, Chiali, Tainan, Taiwan; ^6^Department of Nursing, Min-Hwei Junior College of Health Care Management, Tainan, Taiwan

**Keywords:** resuscitation orders, frail elderly, intensive care units, ventilators, respiratory failure

## Abstract

**Objective:**

As patient life expectancy has increased and people are living longer than before, the rate of mechanical ventilation among elderly patients in the intensive care unit has increased. Older patients who receive mechanical ventilation and have multiple comorbidities are more likely to have a do not resuscitate order than are younger patients with fewer comorbidities. The aim of our study was to describe the patient characteristics and predictive factors of do not resuscitate orders during hospitalization among elderly patients who received ventilation in the intensive care unit.

**Methods:**

This was a retrospective review of the electronic medical records of patients in the intensive care unit of a teaching hospital in southern Taiwan. We enrolled patients admitted to the general intensive care unit from January 1, 2018, to September 31, 2020, and patients older than 80 years who experienced respiratory failure, were intubated and received mechanical ventilation. We analyzed patient demographics, disease severity during hospitalization and comorbidities. If a patient had multiple admissions to the intensive care unit, only the first admission was recorded.

**Results:**

Of the 305 patients over 80 years of age with respiratory failure who were intubated and placed on a ventilator, 66 were excluded because of incomplete data, and 13 were excluded because they had already signed a do not resuscitate order prior to admission to the hospital. Ultimately, 226 patients were included in this study. A higher acute physiology and chronic health evaluation II score (>30) was also associated with an increased likelihood of a do not resuscitate order (odds ratio (OR) = 3.85, 95% CI = 1.09–13.62, *p* = 0.0362). Patients who had acute kidney injury or cerebrovascular accident were more likely to have a do not resuscitate order (OR = 2.74, 95% CI = 1.03–7.28, *p* = 0.0428 and OR = 7.32, 95% CI = 2.02–26.49, *p* = 0.0024, respectively).

**Conclusion:**

Our study showed that older age, greater disease severity, and certain critical interventions were associated with a greater propensity for do not resuscitate orders, which is crucial for understanding patient preferences and guiding end-of-life care discussions. These findings highlight the importance of clinical severity and specific health events in predicting end-of-life care preferences in older patient groups.

## Background

Patients who receive care in the intensive care unit (ICU) are considered critical with severe disease. Due to improvements in medical care and healthier lifestyles, life expectancy and age-related diseases have increased. An increasing number of elderly patients are admitted to the ICU. A study conducted among very old patients (aged ≥80 years) in Bahrain (1) in which Do Not Resuscitate (DNR) orders were not implemented in their hospital due to lack of policy revealed that the outcomes of this population were poor. The in-hospital mortality rate was 96.67% among these elderly individuals. After the first cardiopulmonary resuscitation (CPR), 57.78% of patients died immediately. The one-year survival rate was only 1.11% in that study (1). Another study conducted in a medical center in southern Taiwan (2) enrolled 262 patients with DNR orders after ICU admission and revealed that older patients or patients with malignancies were more likely to have DNR orders than were those without malignancies or younger people. There is a paucity of information on the characteristics of elderly patients (aged ≥80 years) with respiratory failure who receive mechanical ventilation and are admitted to the ICU with a DNR order. With an increasing number of elderly patients and critically ill patients admitted to the ICU and awareness of the optimal quality of life, end-of-life support is needed to ease distressing symptoms (3–7) and provide end-of-life care in the ICU (8). Unfortunately, many patients die in the ICU, highlighting the critical need for excellent end-of-life care as a key component of services in these units (9).

Physicians should be well versed in end-of-life care, which includes understanding both the practical and ethical dimensions, as well as utilizing a mix of pharmacological and nonpharmacological strategies to reduce distress related to the process of dying. It is becoming increasingly important to examine the characteristics of critically ill elderly patients (≥ 80 years) who receive mechanical ventilation, are admitted to the ICU and do not receive CPR. The aim of our study was to describe the clinical features and predictive factors of DNR orders after hospitalization among elderly patients who received mechanical ventilation in the ICU.

## Materials and methods

This study was conducted in the ICU of a teaching hospital in southern Taiwan. The ICU has a total of 21 beds, with 85% of patients aged older than 65 years. Approximately 70% of these patients are residents of long-term care facilities, with an average hospital stay of 5 days. A retrospective study was performed among patients ≥80 years of age who experienced respiratory failure, were intubated and received mechanical ventilation. The study was approved by the Institutional Review Board (IRB) of Chi Mei Medical Center (10911-J02). Informed consent for this retrospective cohort study was waived in accordance with the national legislation and the institutional requirements.

### Study subjects and setting

This was a retrospective review of electronic medical records (EMRs). Data collection was carried out by searching the EMRs of patients admitted to the general ICU from January 1, 2018, to September 31, 2020.

In this study, we collected and statistically analyzed patient demographic information, including age, sex, height, weight, and the source of admission (directly from the emergency department or transferred from a general ward). We also analyzed the following patient data: presence of cancer, Glasgow Coma Scale (GCS) score, Acute Physiology and Chronic Health Evaluation (APACHE) II score, use of bilevel positive airway pressure (BiPAP) before intubation, duration of mechanical ventilation, use of vasopressors, in-hospital mortality, receipt of hemodialysis in the ICU, placement of a nasogastric tube, placement of a urinary catheter, bedridden status before ICU admission, and the Charlson Comorbidity Index (CCI) during hospitalization. The recorded comorbidities included diabetes mellitus (DM), hypertension (HTN), dyslipidemia, end-stage renal disease, dementia or parkinsonism, cerebrovascular accident, atrial fibrillation, coronary artery disease, chronic obstructive pulmonary disease, history of pneumonia, and history of respiratory failure. The primary reasons for ICU admission were categorized as infection, systemic inflammatory response syndrome (SIRS) of non-infectious origin, acute kidney failure, brain injury resulting from an accident, out-of-hospital or in-hospital cardiac arrest, and cardiovascular diseases.

If a patient had multiple admissions to the ICU, only the first admission was recorded. Of the 305 patients over 80 years of age with respiratory failure who were intubated and placed on a ventilator, 66 were excluded because of incomplete data, and 13 were excluded because they had already signed a DNR order prior to admission to the hospital. Ultimately, 226 patients were included in this study.

### Statistical analyses

All the statistical analyses were performed using SPSS version 21.0 (IBM Corp., Armonk, NY, United States). For the descriptive statistical analysis, continuous variables are expressed as the means and standard deviations (SDs), and categorical variables are presented as frequencies and percentages. In addition, the distributions of patients with DNR and without DNR were compared using Student’s t test for continuous variables and Pearson’s chi-square test for categorical variables. To determine the association between potential factors and DNR orders, logistic regression was used to estimate odds ratios (ORs) with 95% confidence intervals (CIs). Subgroup analysis was also conducted to determine the effects of potential confounding factors. A *p* value <0.05 indicated statistical significance.

## Results

The demographic and patient characteristics of elderly patients (≥ 80 years) and the clinical features of 226 patients admitted to the ICU, including 90 patients with DNR orders and 136 patients without DNR orders, are presented in Table 1. The mean age was 86.19 ± 4.24 years, and 128 (56.64%) patients were male. The average body mass index (BMI) was 21.90 ± 4.35. Significant differences were observed in the age distribution between the DNR and non-DNR groups. Patients without a DNR order were younger (<85 and 85 ~ 90) than were those with a DNR order (*p* = 0.0238).

**Table 1:** Demographic characteristics of the enrolled patients.

	All (*n* = 226)	With DNR order (*n* = 90)	Without DNR order (*n* = 136)	*p*-value
Age, mean ± SD	86.19 ± 4.24	86.84 ± 4.57	85.76 ± 3.96	0.0610
Age group, *n* (%)				
<85	92 (40.71)	32 (35.56)	60 (44.12)	0.0238*
85 ~ 90	91 (40.27)	33 (36.67)	58 (42.65)	
>90	43 (19.03)	25 (27.78)	18 (13.24)	
Sex, n (%)				
Male	128 (56.64)	55 (61.11)	73 (53.68)	0.2696
Female	98 (43.36)	35 (38.89)	63 (46.32)	
Height (m)	1.58 ± 0.09	1.58 ± 0.10	1.58 ± 0.08	0.6419
Body weight (kg)	54.60 ± 11.40	54.85 ± 11.27	54.43 ± 11.52	0.7903
BMI, mean ± SD	21.90 ± 4.35	21.89 ± 4.09	21.90 ± 4.53	0.9837
BMI group, n (%)				
<18.5	49 (21.68)	22 (24.44)	27 (19.85)	0.6599
18.5 ~ 24.0	110 (48.67)	41 (45.56)	69 (50.74)	
≥24.0	67 (29.65)	27 (30.00)	40 (29.41)	

The data are presented as the means ± standard deviations (SDs) for continuous variables and frequencies (percentages, %) for categorical variables. *Fisher’s exact test *p* < 0.05.

BMI: body mass index.

Table 2 shows the clinical characteristics and disease severity of the study subjects. The patients were admitted to the ICU most often from the emergency department (*n* = 145, 64.16%), and 81 (35.84%) patients were admitted from the general ward. The mean GCS and APACHE II scores were 8.27 ± 3.24 and 20.74 ± 8.07, respectively. Seventeen (7.52%) patients received BiPAP before intubation. One hundred fifty-six (69.03%) patients received mechanical ventilation for ≥ days. A total of 99 (43.81%) patients died in the hospital. There were 30 (13.27%), 73 (32.30%), and 18 (7.96%) patients who received inotropic agents, including dopamine, norepinephrine and vasopressin, respectively. There were 19 (8.41%) patients who received hemodialysis. The mean CCI score (±SD) was 2.24 (±1.66). Clinical characteristics revealed that the majority of patients had a GCS score of 9–15 (53.98%) and an APACHE II score less than or equal to 30 (91.15%). However, patients with a DNR order had a significantly greater proportion of APACHE II scores greater than 30 (14.44% vs. 5.15%, *p* = 0.0160) and a greater rate of norepinephrine use (45.56% vs. 23.53%, *p* = 0.0005). The mortality rate was significantly greater in the DNR group (66.67% vs. 28.68%, *p* < 0.0001). A significant proportion of patients with a DNR order had comorbidities (> 60%), including HTN (82.22%), a history of pneumonia (63.33%) and a history of respiratory failure (62.22%). The three most common reasons for ICU admission among patients with a DNR order were infection (40%), SIRS of noninfectious origin (31.11%), and acute kidney injury (23.33%).

**Table 2:** Clinical characteristics and disease severity of the enrolled patients.

	All (*n* = 226)	With DNR order (*n* = 90)	Without DNR order (*n* = 136)	*p*-value
Admission source, *n* (%)				
Emergency department	145 (64.16)	62 (68.89)	83 (61.03)	0.2277
Wards	81 (35.84)	28 (31.11)	53 (38.97)	
Malignancy, n (%)	53 (23.45)	20 (22.22)	33 (24.26)	0.7228
GCS score, mean ± SD	8.27 ± 3.24	7.92 ± 3.35	8.49 ± 3.16	0.1956
GCS group, *n* (%)				
9–15	122 (53.98)	45 (50.00)	77 (56.62)	0.3285
3–8	104 (46.02)	45 (50.00)	59 (43.38)	
APACHE II score, mean ± SD	20.74 ± 8.07	21.63 ± 8.54	20.15 ± 7.71	0.1778
APACHE II group, *n* (%)				
≤30	206 (91.15)	77 (85.56)	129 (94.85)	**0.0160**
>30	20 (8.85)	13 (14.44)	7 (5.15)	
Used BiPAP, n (%)	17 (7.52)	6 (6.67)	11 (8.09)	0.6916
Ventilator duration, *n* (%)				
≤7 days	156 (69.03)	55 (61.11)	101 (74.26)	**0.0363**
>7 days	70 (30.97)	35 (38.89)	35 (25.74)	
Inotropic agent, *n* (%)	86 (38.05)	46 (51.11)	40 (29.41)	**0.0010**
Dopamine, *n* (%)	30 (13.27)	15 (16.67)	15 (11.03)	0.2214
Norepinephrine, *n* (%)	73 (32.30)	41 (45.56)	32 (23.53)	**0.0005**
Vasopressin, *n* (%)	18 (7.96)	10 (11.11)	8 (5.88)	0.1552
Mortality, *n* (%)	99 (43.81)	60 (66.67)	39 (28.68)	**<0.0001**
Hemodialysis in the ICU, *n* (%)	19 (8.41)	10 (11.11)	9 (6.62)	0.2334
Nasogastric tube, *n* (%)	49 (21.68)	22 (24.44)	27 (19.85)	0.4122
Foley catheter, *n* (%)	50 (22.12)	23 (25.56)	27 (19.85)	0.3120
Bedridden, *n* (%)	38 (16.81)	18 (20.00)	20 (14.71)	0.2975
CCI, mean ± SD	2.24 ± 1.66	2.21 ± 1.55	2.26 ± 1.73	0.8380
CCI group, *n* (%)				
0	32 (14.16)	11 (12.22)	21 (15.44)	0.7565
1–2	103 (45.58)	43 (47.78)	60 (44.12)	
>2	91 (40.27)	36 (40.00)	55 (40.44)	
Comorbidities, *n* (%)				
Diabetes	85 (37.61)	39 (43.33)	46 (33.82)	0.1485
Hypertension	182 (80.53)	74 (82.22)	108 (79.41)	0.6014
Dyslipidemia	20 (8.82)	10 (11.11)	10 (7.35)	0.3302
End-stage renal disease	52 (23.01)	20 (22.22)	32 (23.53)	0.8192
Dementia/parkinsonism	45 (19.91)	19 (21.11)	26 (19.12)	0.7133
Cerebrovascular accident	66 (29.20)	27 (30.00)	39 (28.68)	0.8304
Atrial fibrillation	84 (37.17)	36 (40.00)	48 (35.29)	0.4736
Coronary artery disease	66 (29.20)	23 (25.56)	43 (31.62)	0.3265
History of pneumonia	142 (62.83)	57 (63.33)	85 (62.50)	0.8990
COPD	30 (13.27)	9 (10.00)	21 (15.44)	0.2379
History of respiratory failure	129 (57.08)	56 (62.22)	73 (53.68)	0.2039
*Reasons for ICU admission*				
Infection, n (%)	85 (37.61)	36 (40.00)	49 (36.03)	0.5464
SIRS, n (%)	62 (27.43)	28 (31.11)	34 (25.00)	0.3135
Acute kidney injury, *n* (%)	41 (18.14)	21 (23.33)	20 (14.71)	0.0994
Traumatic brain injury, *n* (%)	12 (5.31)	6 (6.67)	6 (4.41)	0.5487
Cardiac arrest, *n* (%)	21 (9.29)	11 (12.22)	10 (7.35)	0.2171
Cerebrovascular accident, *n* (%)	19 (8.41)	13 (14.44)	6 (4.41)	**0.0078**

The data are presented as the means ± standard deviations (SDs) for continuous variables and frequencies (percentages, %) for categorical variables.

APACHE II, Acute Physiology and Chronic Health Evaluation II; BiPAP, bilevel positive airway pressure; CCI, Charlson Comorbidity Index; COPD, chronic obstructive pulmonary disease; DNR, Do Not Resuscitate; GCS, Glasgow Coma Scale; ICU, intensive care unit; SIRS, systemic inflammatory response syndrome.

Table 3 shows potential predictors of DNR orders according to the logistic regression model. According to the multivariate regression, patients older than 90 years had greater odds of having a DNR order than did those younger than 85 years (OR = 4.45, 95% CI = 1.66–11.90, *p* = 0.003). A higher APACHE II score (>30) was also associated with an increased likelihood of a DNR order (OR = 3.85, 95% CI = 1.09–13.62, *p* = 0.0362). Additionally, patients who had acute kidney injury or cerebrovascular accident were more likely to have a DNR order (OR = 2.74, 95% CI = 1.03–7.28, *p* = 0.0428 and OR = 7.32, 95% CI = 2.02–26.49, *p* = 0.0024, respectively).

**Table 3:** Predictors of DNR orders according to the logistic regression model.

	Univariate OR (95% CI)	*p*-value	Multivariate OR (95% CI)	*p*-value
Age group (reference: <85)				
85 ~ 90	1.07 (0.58–1.96)	0.8342	1.02(0.46,2.28)	0.9637
>90	2.60 (1.24–5.47)	**0.0115**	4.45(1.66,11.90)	**0.0030**
Sex (reference: female)	1.36 (0.79–2.33)	0.2701	1.13(0.55,2.29)	0.7416
BMI group (reference: 18.5 ~ 24.0)				
<18.5	1.37 (0.69–2.71)	0.3648	1.69(0.70,4.10)	0.2480
≧24.0	1.14 (0.61–2.12)	0.6882	1.01(0.44,2.30)	0.9846
Admission source: ER (reference: wards)	1.41 (0.81–2.49)	0.2286	1.33(0.63,2.81)	0.4554
Malignancy	0.89 (0.47–1.68)	0.7228	0.85(0.29,2.52)	0.7696
GCS score	0.95 (0.87–1.03)	0.1953		
GCS group: ≦8 (reference: GCS: 9–15)	1.31 (0.77–2.23)	0.3289	0.57(0.26,1.26)	0.1634
APACHE II score > 30 (reference: ≦30)	3.11 (1.19–8.14)	**0.0207**	3.85(1.09,13.62)	**0.0362**
Used BiPAP	0.81 (0.29–2.28)	0.6921	0.88(0.23,3.36)	0.8548
Ventilator duration >7 days (reference: ≦7 days)	1.84 (1.04–3.25)	**0.0373**	1.93(0.91,4.12)	0.0873
Any vasopressor	2.51 (1.44–4.37)	**0.0011**	1.45(0.29,7.16)	0.6514
Dopamine	1.61 (0.75–3.49)	0.2243	1.55(0.49,4.97)	0.4572
Norepinephrine	2.72 (1.53–4.83)	**0.0006**	2.67(0.63,11.28)	0.1814
Vasopressin	2.00 (0.76–5.28)	0.1617	1.33(0.31,5.70)	0.6992
Hemodialysis in the ICU	1.76 (0.69–4.53)	0.2382	0.59(0.13,2.73)	0.4965
Nasogastric tube	1.31 (0.69–2.48)	0.4129	0.51(0.06,4.24)	0.5339
Foley catheter	1.39 (0.74–2.61)	0.3130	2.26(0.27,18.98)	0.4531
Bedridden	1.45 (0.72–2.92)	0.2991	1.29(0.43,3.86)	0.6526
CCI group (references: 0)				
1–2	1.37 (0.60–3.13)	0.4580	2.04(0.58,7.21)	0.2671
>2	1.25 (0.54–2.90)	0.6039	2.52(0.37,17.39)	0.3489
Comorbidities				
Diabetes	1.50 (0.87–2.59)	0.1494	1.36(0.58,3.17)	0.4762
Hypertension	1.20 (0.61–2.37)	0.6017	0.82(0.35,1.97)	0.6633
Dyslipidemia	1.58 (0.63–3.95)	0.3333	2.02(0.58,7.10)	0.2726
End-stage renal disease	0.93 (0.49–1.75)	0.8200	0.66(0.22,1.98)	0.4634
Dementia/parkinsonism	1.13 (0.58–2.20)	0.7134	0.84(0.34,2.09)	0.7109
Cerebrovascular accident	1.07 (0.59–1.91)	0.8300	0.82(0.35,1.91)	0.6447
Atrial fibrillation	1.22 (0.71–2.12)	0.4738	1.36(0.58,3.17)	0.4814
Coronary artery disease	0.74 (0.41–1.35)	0.3273	0.47(0.18,1.21)	0.1181
History of pneumonia	1.04 (0.60–1.80)	0.8991	0.84(0.37,1.89)	0.6671
COPD	0.61 (0.27–1.40)	0.2414	0.45(0.15,1.34)	0.1502
History of respiratory failure	1.42 (0.83–2.45)	0.2046	0.93(0.40,2.17)	0.8646
Reasons for ICU admission				
Infection/sepsis	1.18 (0.68–2.05)	0.5465	0.56(0.18,1.78)	0.3238
SIRS	1.36 (0.75–2.45)	0.3142	1.02(0.30,3.43)	0.9757
Acute kidney injury	1.77 (0.89–3.49)	0.1019	2.74(1.03,7.28)	**0.0428**
Traumatic brain injury	1.56 (0.53–4.59)	0.4250	2.95(0.63,13.71)	0.1687
Cardiac arrest	1.75 (0.71–4.32)	0.2216	1.29(0.39,4.32)	0.6781
Cerebrovascular accident	3.66 (1.34–10.02)	**0.0116**	7.32(2.02,26.49)	**0.0024**

APACHE II, Acute Physiology and Chronic Health Evaluation II; BiPAP, bilevel positive airway pressure; BMI, body mass index; CCI, Charlson Comorbidity Index; COPD, chronic obstructive pulmonary disease; DNR, Do Not Resuscitate; ER, emergency department; GCS, Glasgow Coma Scale; ICU, intensive care unit; SIRS, systemic inflammatory response syndrome.

The results of the sex-stratified subgroup analysis of different predictors of DNR orders using a logistic regression model are presented in Table 4. According to our sex-specific analysis, being older than 90 years of age significantly predicted DNR orders for males (OR = 3.05, *p* = 0.0283). Males with an APACHE II score greater than 30 had significantly greater odds of having a DNR order, with an OR of 3.96 (95% CI = 1.11–14.09, *p* = 0.0338). A cerebrovascular accident significantly increased the likelihood of having a DNR order for both males (OR = 3.84, *p* = 0.0414) and females, although it was marginally significant for the latter (OR = 5.23, *p* = 0.0675).

**Table 4:** Predictors of DNR orders by sex according to the logistic regression model.

	Male		Female	
	OR (95% CI)	*p*-value	OR (95% CI)	*p*-value
Age group (reference: <85)				
85–90	1.28 (0.55–2.99)	0.5677	0.88 (0.32–2.40)	0.7954
>90	3.05 (1.13–8.25)	**0.0283**	2.90 (0.80–10.45)	0.1044
APACHE II score > 30 (reference: ≦30)	3.96 (1.11–14.09)	**0.0338**	2.08 (0.37–11.87)	0.4091
Ventilator duration >7 days (reference: ≦7 days)	1.22(0.55–2.71)	0.6233	3.32 (1.26–8.74)	**0.0152**
Cerebrovascular accident	3.84 (1.05–13.95)	**0.0414**	5.23 (0.89–30.75)	0.0675

APACHE II, Acute Physiology and Chronic Health Evaluation II; DNR, Do Not Resuscitate; OR, odds ratio.

However, for females, requiring a ventilator for more than 7 days was a significant predictor of a DNR order (OR = 3.32, *p* = 0.0152).

Table 5 shows the predictors of DNR orders according to age group (<85 years, 85–90 years, and > 90 years). The 85–90 age group had a greater likelihood of a DNR order (OR = 4.40, 95% CI = 0.94–20.61), and the result was significant (*p* = 0.0604). In addition, a history of cerebrovascular accident was a significant predictor of a DNR order in the 85–90 age group (OR = 4.98, 95% CI = 1.11–22.28, *p* = 0.0356).

**Table 5:** Predictors of DNR orders by age group according to the logistic regression model.

	<85 years		85–90 years		>90 years	
	OR (95% CI)	*p-*value	OR (95% CI)	*p-*value	OR (95% CI)	*p-*value
APACHE II score > 30 (reference: ≦30)	2.22 (0.41–11.91)	0.3541	4.40 (0.94–20.61)	0.0604	3.97 (0.40–39.06)	0.2367
Ventilator duration >7 days (reference: ≦7 days)	1.64 (0.64–4.21)	0.3026	2.16 (0.84–5.56)	0.1111	2.27 (0.48–10.70)	0.3017
Cerebrovascular accident	3.21 (0.69–15.04)	0.1381	4.98 (1.11–22.28)	**0.0356**	NA*	NA*

*NA: not available.

APACHE II, Acute Physiology and Chronic Health Evaluation II; DNR, Do Not Resuscitate; OR: odds ratio.

Table 6 shows the predictors of DNR orders stratified by GCS score. The analysis was separated into two categories: patients with a GCS score greater than 8 and those with a GCS score of 8 or less. Patients with a GCS score ≤ 8 and an APACHE II score greater than 30 had a 3.49-fold (95% CI: 1.00–12.16, *p* = 0.0494) greater odds of signing DNR orders than patients with an APACHE II score ≤ 30. In addition, in this subgroup, ICU admission due to cerebrovascular accident significantly increased the likelihood of DNR orders, with an OR of 6.64 (1.58–27.87, *p* = 0.0097).

**Table 6:** Predictors of DNR orders by GCS score according to the logistic regression model.

	GCS score > 8		GCS score ≤ 8	
	OR (95% CI)	*p-*value	OR (95% CI)	*p-*value
Age group (reference: <85)				
85–90	1.58 (0.67–3.72)	0.2998	0.60 (0.22–1.60)	0.3049
>90	2.62 (0.88–7.79)	0.0827	2.72 (0.84–8.73)	0.0937
APACHE II score > 30 (reference: ≦30)	3.47 (0.56–21.61)	0.1825	3.49 (1.00–12.16)	**0.0494**
Ventilator duration >7 days (reference: ≦7 days)	2.56 (1.09–6.05)	**0.0316**	1.38 (0.57–3.35)	0.4731
Cerebrovascular accident	2.61 (0.52–12.96)	0.2420	6.64 (1.58–27.87)	**0.0097**

APACHE II, Acute Physiology and Chronic Health Evaluation II; DNR, Do Not Resuscitate; GCS, Glasgow Coma Scale; OR, odds ratio.

For patients with a GCS score > 8, a ventilation duration greater than 7 days was a significant predictor of DNR orders, with an OR of 2.56 (1.09–6.05, *p* = 0.0316).

## Discussion

This study investigated the characteristics of elderly (>80 years) patients who were admitted to the ICU and received mechanical ventilation and signed DNR orders. According to our study, a higher APACHE II score, longer duration of ventilator use, inotropic agent use and admission to the ICU due to cerebrovascular accident were significant predictors of DNR orders. Patients with these characteristics were more likely to have a DNR order. These findings highlight the importance of considering patient-specific factors in elderly patients with respiratory failure in the ICU when discussing and documenting end-of-life care preferences.

### DNR orders and disease severity

In a study, most people favored improving their quality of life for the time they had left, ranging from 57 to 81%. Only a minority (2–6%) of people said extending life was important, regardless of their health status (10).

Comparatively, a retrospective cohort study by Wu et al. (11) was carried out in a geriatric ward at a tertiary hospital in southern Taiwan from 2018 to 2019. Their study included 337 hospitalized elderly patients aged >65 years in the geriatric ward and identified age, poor nutritional status, lower albumin levels, lower CCI, and ICU transfer as independent factors associated with DNR orders. Our study also highlighted age as a critical factor, with older patients being more likely to have a DNR order. This finding is consistent across different care settings in the ward or ICU, reflecting a broader recognition of the limited benefits of aggressive treatments or poor prognosis in older patients and of more conservative care preferences among older patients. Additionally, both studies underscore the importance of clinical severity and health status in DNR decisions. The presence of major comorbidities, as indicated by the CCI in the study by Wu et al. (11), and higher APACHE II scores in our research suggest that a more advanced disease state influences DNR orders.

In critical care research, illness severity scores are often used for risk adjustment and mortality prediction, with the APACHE II being a prominent tool that emphasizes physiological abnormalities. In contrast to this approach, administrative data often utilize risk adjustment systems such as the CCI, which are exclusively based on the existence of comorbidities. An earlier study (12) used clinical data to make a comparison with hospital outcome statistics. This study applied multiple regression analysis to determine the accuracy of the APACHE II score and the CCI for predicting in-hospital mortality among adult patients in the ICU. Additionally, we investigated how well the CCI performed on its own and when combined with the APACHE II score for predicting hospital mortality. Their results showed, as anticipated, that the APACHE II score accurately predicted in-hospital mortality.

Our study also emphasized the role of specific clinical interventions and conditions, such as ventilator duration, inotropic agent use and reason for admission to the ICU. In contrast, Wu et al. highlighted the importance of nutritional status and albumin levels, which are more pertinent to the geriatric ward context. This difference might reflect the varying patient populations and the distinct focus areas within different care settings. While ICU admissions often involve more acute and severe conditions, geriatric wards typically manage chronic and deteriorating health states, where nutritional status and general well-being play a more prominent role.

The difference in the emphasis on clinical severity versus nutritional status and albumin levels might result from the different patient populations and settings, suggesting that some factors, such as age and disease severity, are universally significant. Understanding these predictors is crucial for clinicians to initiate timely and appropriate discussions about end-of-life care preferences with patients and their families. Recognizing the roles of age, clinical severity, and health status can help tailor these conversations to ensure that they are both relevant and sensitive to the patient’s condition and likely trajectory.

### DNR orders and cerebrovascular disease

In a comprehensive Taiwanese study, using data from the Taiwan Stroke Registry, researchers examined hospitalized stroke patients across 64 hospitals from 2006 to 2020 (13). They employed a two-level random effects model to determine the factors associated with the issuance of DNR orders. Their results indicated that among these patients, those with acute ischemic stroke most frequently had DNR orders, followed by individuals suffering from intracerebral hemorrhage. There was a noticeable increase in DNR orders among stroke patients throughout the 14-year study period. Their study also highlighted that in ischemic stroke scenarios, female patients tended to have DNR orders more often. Furthermore, the study highlighted the important role of hospital characteristics in influencing the use of DNR orders. Our study had similar results. Elderly patients with respiratory failure who were admitted to the ICU due to cerebrovascular accident tended to have DNR orders. In addition, females who were admitted to the ICU with cerebrovascular accident and respiratory failure had a higher probability of having a DNR order (OR = 5.23), but this difference was marginally significant because of the small sample size in our study.

Moreover, both studies noted the significance of clinical severity in DNR decisions. A higher APACHE II score in our study and a higher National Institutes of Health Stroke Scale score in the Yeh et al. (13) study were associated with an increased likelihood of DNR orders, underscoring the role of disease severity in end-of-life decision-making. They showed the impact of hospital characteristics, with patients treated at religious hospitals and medical centers showing different propensities for DNR orders. This difference might reflect the varying patient populations and the distinct focus areas within different care settings. Taiwan’s National Health Insurance (NHI) boasts one of the world’s lowest administrative expenses. This system allows Taiwanese residents to consult with any doctor of their choosing without needing a referral. Additionally, they have the freedom to directly seek care at any hospital, regardless of its level, according to their preference (14). If patients or their families request more aggressive treatment, they can visit a medical center, and elderly patients residing in long-term care facilities with multiple comorbidities tend to receive conservative treatment at regional or district hospitals.

### DNR orders and the ICU

A retrospective analysis of patients with an established DNR status upon admission to the ICU of a single hospital was performed, covering a period of 18 months (15). A total of 35 patients qualified for the study. The predominant causes for ICU admission were respiratory distress (54.2%) and sepsis (45.7%). Among these patients, 16 (45.7%) died, in contrast to the overall ICU mortality rate of 5.4% during the same timeframe. In our study, the enrolled elderly patients with DNR orders had a 66.7% higher mortality rate than did those in the study by Saha et al. (15).

Our study suggested that elderly patients with DNR orders in the ICU have complex needs and often face a higher risk of mortality, indicating the need for healthcare providers to consider the implications of DNR status thoroughly and to engage in comprehensive discussions with patients and families about end-of-life care preferences. Regular and repeated discussions with patients and families about end-of-life wishes, as well as the integration of palliative care services, are needed to support DNR patients effectively.

The high mortality rates underscore the importance of aligning patient care with patient values and preferences to identify factors predicting DNR orders, potentially aiding in early discussions and decision-making about end-of-life care.

### DNR orders and life-sustaining treatments

Chang et al. (16) conducted a prospective descriptive and correlational study, providing evaluative insight into the impact of DNR orders on patient care and finding that patients with a DNR order were less frequently administered life-sustaining treatments than were those without such an order. DNR orders have an impact on life-supporting therapies. Patients who have a DNR order before admission often receive fewer life-sustaining treatments. Our DNR patients were treated more often with inotropic agents and aggressive treatment than patients without DNR orders because our patients requested DNR orders during hospitalization, and DNR requests resulted from poor clinical treatment efficacy after aggressive life-sustaining treatments such as inotropic agents and hemodialysis.

These findings reinforce the critical nature of DNR discussions and the need for healthcare professionals to have these conversations with clear understanding from patients or their surrogates after little benefit is observed from treatment. The choice to either withhold or discontinue medical treatments should be a collaborative process involving both the healthcare providers and the patients or their designated representatives. This decision-making process should center on the patient’s personal values and treatment preferences. It is crucial that each decision reflect the patient’s general prognosis, the potential effectiveness of the specific treatment, the balance of its benefits and drawbacks for the patient, and the overarching objectives of the patient’s care plan.

The implications of our findings are important for clinical practice. Understanding the predictors of DNR orders can help healthcare providers identify patients who might benefit from early discussions about their care preferences. This approach is particularly crucial for ensuring that end-of-life care aligns with patient values and reduces unnecessary and potentially harmful interventions. The differences underline the importance of considering the local context, ethics, culture, and patient demographics. We need clear guidelines and communication strategies to navigate the complexities of end-of-life care in ICUs to ensure that ethical, patient-centered care at the end of life and patient preferences are accurately represented and honored.

### Limitations

This study has the following limitations. First, this was a retrospective observational study in a single hospital in Taiwan, and healthcare access bias may exist. Our data may not represent data from other medical centers or patients in other countries. Second, we analyzed only patients admitted to the ICU; some elderly patients with critical illness may have refused to be admitted to the ICU and received treatment in the general ward, which may have led to confounding. Third, we did not evaluate patient condition after discharge from the hospital. We did not have long-term follow-up data; thus, we were not able to analyze mortality among elderly patients after discharge. Furthermore, cultural, social, and individual preferences, which play a substantial role in end-of-life decisions, were not directly addressed. The retrospective study design limited our ability to infer causality. Additionally, our patient population was specific to a Taiwanese ICU setting, which might limit the generalizability of our findings to other settings or populations.

This was a retrospective observational study in a single hospital in Taiwan, which may lead to the presumed bias due to healthcare access difference. However, in Taiwan, patients have had the legal right to request a DNR since June 7, 2000. Based on the share decision-making with healthcare providers, patients, and their families, the equitable treatment and informed consent of DNR was obtained by patients or their legally authorized representatives for the end-of-life care. Therefore, the presumed bias may be existed in our research findings within the context of the Taiwanese healthcare system. It’s important to note that patients in our study may not be fully represented in other countries.

The aim of our study was to determine the factors influencing DNR orders after hospitalization among elderly patients admitted to the ICU who received mechanical ventilation. Future research should aim to prospectively validate these predictors in a more diverse patient population. Additionally, qualitative studies exploring the reasons behind different preferences for DNR orders could provide deeper insights into how patients and their families make these complex decisions. Understanding these nuances is crucial for tailoring end-of-life discussions and care to meet individual needs.

## Conclusion

In conclusion, elderly patients had a high mortality rate after admission to the intensive care unit. Our study revealed that the following factors were significant predictors of a do not resuscitate order after hospitalization: an acute physiology and chronic health evaluation II score > 30, >7 days of ventilation, inotropic agent use and admission to the intensive care unit due to cerebrovascular accident. Understanding these predictors is crucial for clinicians to initiate timely and appropriate discussions about end-of-life care preferences with patients and their families. If a poor prognosis can be predicted from a clinical scenario, a do not resuscitate order and other supportive treatments should be initiated early during the intensive care unit stay. Our study adds to the growing body of literature on do not resuscitate orders in intensive care unit settings. By identifying key predictors of do not resuscitate orders, we hope to contribute to more personalized, value-aligned care for critically ill patients at the end of life.

## Data availability statement

The raw data supporting the conclusions of this article will be made available by the authors, without undue reservation.

## Ethics statement

The studies involving humans were approved by the Institutional Review Board (IRB) of Chi Mei Medical Center (10911-J02). Informed consent for this retrospective cohort study was waived in accordance with the national legislation and the institutional requirements. The studies were conducted in accordance with the local legislation and institutional requirements. Written informed consent for participation was not required from the participants or the participants’ legal guardians/next of kin in accordance with the national legislation and institutional requirements.

## Author contributions

P-JC: Conceptualization, Data curation, Writing – original draft. C-HH: Data curation, Formal analysis, Methodology, Writing – original draft. Y-JL: Formal analysis, Visualization, Writing – original draft. M-HC: Conceptualization, Investigation, Writing – original draft. K-ML: Conceptualization, Writing – original draft, Writing – review & editing, Data curation, Methodology, Resources.
